# Still human: Presence, oncology, and artificial intelligence

**DOI:** 10.1093/oncolo/oyag199

**Published:** 2026-05-20

**Authors:** Burcu Ulaş Kahya

**Affiliations:** Department of Medical Oncology, Karaman Training and Research Hospital, Karaman, Türkiye

It was almost three in the morning. I was sitting with a cup of tea that had gone cold, scrolling through my phone, when I came across something that made me stop. A research group in Japan had built an artificial intelligence that could hold conversations about Buddhist philosophy: about impermanence, about suffering, about letting go.

I sat with it for a while. There was something unsettling about it that I couldn’t quite name. Buddhism, of all things, is built on the idea that suffering has to be lived through—not just understood. And here was a system that had never lost anyone, never sat at a bedside, never watched a family quietly fall apart in a hospital corridor, speaking about all of it with great fluency and confidence.

I thought: this is where we are. We can now simulate the surface of almost anything.

Then I thought about my patients. About who I would see in the morning. And the unease about the machine suddenly felt smaller.

People sometimes ask me whether oncology is depressing. I never quite know how to answer. Yes, I see suffering every day. I see fear on faces, families trying to hold themselves together in waiting rooms, people receiving news they were not ready for. I carry these experiences home far more deeply than I usually acknowledge.

But I also see something that I think is rare in modern life: people being completely, unguardedly honest. When you are facing a serious illness, the small pretenses tend to fall away. People say what they actually mean. They ask questions the rest of us push aside for later. There is a kind of depth in those rooms that I have not found anywhere else. Bearing witness is not merely a burden; it is a profound privilege—one I lean on as a source of purpose during the most challenging days.

One of my patients is 45 years old. In the past decade, he lost his mother, his wife, and his brother—all to cancer. He had always been the caregiver. This time, he was the patient. He knows what this illness looks like from every angle.

Now it is his turn.

At the end of an appointment, he said something quietly, the way a person says something they have already made peace with: “I want to die alone. I don’t want my younger sister and brother to go through it again. I know what watching someone die does to you. I don’t want to be one more thing they carry.”

I did not say anything right away. Some things deserve a moment before you respond.

There was no self-pity in it. No performance. Just a man who had learned, through unbearable repetition, what grief costs—and who was trying, in the only way he had left, to protect the people he loved from paying that cost again. It was, I realized, an act of love. One of the purest I have witnessed.

I thought about the Buddhist AI again. It could probably produce a thoughtful response to what he had told me. It might even sound compassionate.

But it would not know what it means to sit across from a man like him and simply stay. To let what he said land without trying to fix it or redirect it. That does not come from data. It comes from being a person who will also, one day, lose someone. Who will also, one day, be lost.

That is what I keep coming back to, in a world that is getting better and better at imitating human things. Presence cannot be imitated. Remaining present in difficult moments—witnessing suffering unflinchingly—requires a human connection that carries a personal cost. Someone who is not outside the story, but inside it.

Oncologists live inside the story. Every day, whether we think about it that way or not.

Morning came. I put on my coat and went to the hospital. Outside, the city was going about its ordinary life: horns honking, children going to school.

Whatever those in the waiting room needed—information, honesty, or silence—I was certain they required a clinician willing to be truly present.

I am glad this remains a human task.

